# Rainfall distribution variability controls surface but not belowground litter decomposition in a semi-arid shrubland

**DOI:** 10.3389/fpls.2025.1455170

**Published:** 2025-02-05

**Authors:** Yulin Li, Li Cheng, Honglin Yang, Rui Zhang, Zhiying Ning

**Affiliations:** Key Laboratory of Ecological Safety and Sustainable Development in Arid Lands, Northwest Institute of Eco-Environment and Resources, Chinese Academy of Sciences, Lanzhou, China

**Keywords:** decomposition constant, precipitation frequency, precipitation amount, mass loss, mixed effect

## Abstract

**Introduction:**

Rainfall patterns are expected to become increasingly erratic as a result of global climate change, with more intense but less frequent rainfall events leading to an increased occurrence of drought events. This process may lead to significant declines in vegetation cover and subsequent increases in soil erosion, consequently accelerating the bury of detached litter by soil deposition and the mixture of residues from different plant species. Responses of litter decomposition to increasing rainfall variability in distribution and subsequent litter mixing or soil cover have scarcely received attention.

**Methods:**

To fill this gap in our knowledge, we analyzed the influence of rainfall variability, soil cover, and litter mixing on shrub-species litter decomposition in a semi-arid shrubland. We explored the effects of redistributing the frequency and amount of precipitation on surface and belowground decomposition of litter from two separate or mixed predominant shrubs.

**Results:**

Decomposition of belowground litter was consistently higher than that of surface litter over the entire field-incubation process. Mass loss significantly decreased in surface litter but not in belowground litter due to the lower frequency and larger amount of precipitation compared to the control treatment. Furthermore, exclusion of 30% precipitation had no significant effects on decomposition of either surface or belowground litter. We observed stronger synergistic effect for belowground litter mixture relative to surface litter mixture of the two shrubs, especially in the hotter months over the 5-month incubation.

**Discussion:**

These findings support that rainfall variability in terms of distribution pattern rather than in the amount controls the litter decomposition on the soil surface in the semi-arid shrubland. Meanwhile, soil burial or litter mixing have greater effects on litter decomposition, individually or jointly. Together, our results highlight the need to consider rainfall distribution variability and incorporate soil-covering and litter-mixing as driving factors of organic matter turnover in drylands.

## 1 Introduction

The decomposition of plant material in arid and semi-arid ecosystems plays an important role in regulating carbon storage and nutrient cycling because global drylands account for approximately 30% of net primary production (Field et al., 1998; Austin et al., 2004). A notable characteristic of drylands is the relative shortage of natural rainfall which is considered to constrain plant material decomposition (Throop and Archer, 2009). However, a growing body of evidence suggests that responses of litter decomposition are inconsistent with changes in rainfall at the local scale (Yahdjian et al., 2006; Dirks et al., 2010; Li et al., 2016). Some previous observations suggested that litter decomposition rates correlate well (Yahdjian et al., 2006; Brandt et al., 2007) or do not correlate with seasonal or annual precipitation (Vanderbilt et al., 2008; Gallo et al., 2009). This discrepancy was partially attributed to the fact that current studies in drylands have mostly focused on the effects of cumulative rainfall rather than rainfall variability, which is one of the dominant components of climate change (Joly et al., 2019). Rainfall variability is usually exhibited as a complex pattern including annual or seasonal variations in the total amount of the precipitation, intensity, interval, and timing of rainfall events, etc (Smith, 2011; Beier et al., 2012). In terms of a given cumulative precipitation quantity, it is unknown whether the effects of large and infrequent precipitation events on inducing and sustaining litter decomposition are equivalent to smaller but more frequent rainfall events. However, responses of litter decay to rainfall variability scarcely received attention, because of the difficulty in simulating the complexity of natural rainfall regimes in drylands (Joly et al., 2019).

Ongoing global rainfall changes in arid regions are expected to become more variable, with less frequent rainfall events and subsequent increases in the durations of drought (Easterling et al., 2000; IPCC et al., 2013). One of the predictable consequences of high rainfall variability in arid and semi-arid ecosystems is that surface litters and upper soils undergo longer periods of drying before being rewetted by a following rainfall pulse (Austin et al., 2004). These extended durations of dry periods between pulses thereby may constrain litter decomposition by shortening accumulative time that litter has adequate moisture for activities of microbial decomposers and detritivores. Such response patterns have been demonstrated that large and infrequent rainfall pulses reduce litter decomposition (Whitford et al., 1986); but may vary with rainfall pulse sizes, as, for example, has been shown for the non-linear responses of decomposition to increasing cumulative rainfall quantity under low rainfall frequencies (Joly et al., 2017b). However, robust evidence of the interaction between pulse size and frequency on decomposition in drylands remains limited to a very small number of microcosm experiments with restricted scope for generalizability and validity (Whitford et al., 1986; Austin et al., 2009; Joly et al., 2017a, 2019).

Extended durations of dry periods induced by high rainfall variability can have a direct impact on plant growth in drylands, and, hence, can lead to significant declines in vegetation cover and subsequent increases in soil erosion (Austin et al., 2004; Austin, 2011). This process no doubt accelerates the covering of detached litter by soil deposition and the mixing of litters from different plant species (Hewins et al., 2013; Lee et al., 2014), especially under the shrub canopy or in some aeolian accumulation areas (Garner and Steinberger, 1989; Austin, 2011). Several studies have demonstrated that litter mixtures often decompose at a different rate relative to the average rate of the individual litters (Berglund and Ågren, 2012). A synthesized study concluded that 67% of studies on litter mixture decomposition was non-additive, namely, the decomposition rate of the mixture is either faster or slower than the average rate of the individual litters (Gartner and Cardon, 2004). These non-additive effects mainly resulted from accelerated or decelerated decomposition of certain species litters. Simultaneously, decomposition of litter was considered to be accelerated after soil-covering (or soil-mixing) because such process may buffer litter from high frequency moisture oscillations in the soil relative to that on the surface, thereby increasing the opportunity for microbially-mediated decomposition (Hewins et al., 2013; Lee et al., 2014; Joly et al., 2017a). Moreover, moisture oscillation in soil is heavily dependent upon rainfall pulse size and frequency (Austin et al., 2009). Unfortunately, little is currently known about how increases of rainfall variability affects litter decomposition under conditions of soil-covering or litter mixtures, and uncovering these processes can provide new insights into how litter decay responds to ongoing rainfall changes in arid regions.

In this study, we quantified the effects of rainfall variability on surface and belowground (simulated soil-covering) litter decomposition in semi-arid shrubland of northern China. Litters of two dominant shrubs and their litter mixture were incubated in the field, where the frequency and amount of natural rainfall was artificially altered in a growing season. The specific objectives of the present study were to address three guiding research questions: 1) How does increased rainfall variability or decreased rainfall amounts affect dry mass loss of litter in semi-arid shrubland? 2) Does belowground litter decompose faster than surface litter under conditions of simulated soil-covering? 3) If the decomposition rate of the litter mixture is consistent with the sum of the individual litter?

## 2 Materials and methods

### 2.1 Study site

The study was carried out at Naiman Desertification Research Station in Horqin Sand Land, Chinese Academy of Sciences (NDRS, Inner Mongolia, China, 42˚56ˊ26˝N, 120˚43ˊ05˝E, 358 m altitude) Mean annual temperature in the area is 6.4°C, with monthly averages ranging from a minimum of -13.1°C in January to a maximum of 23.7°C in July. The yearly average solar radiation is 5200 MJ/m^2^, while the total yearly sunshine duration is about 2946 h. The mean annual precipitation is 362 mm, nearly 70 - 80% of which falls between April and October. During the growing season, approximately 58.7% of rainfall events are ≤ 5 mm and contribute only 12.6% of the total rainfall amount, while 9.6% of large events with rainfall > 20 mm account for 45.0% of the total rainfall (Yue et al., 2016). The soils are light yellow in color, very infertile, and sandy with a coarse texture and loose structure (Su et al., 2006). In general, the maximum sand content (1 - 0.05 mm) in the soils of the study site is 90%, with organic matter and total N contents of less than 1.0 g kg^-1^ and 0.13 g kg^-1^, respectively (Zhou et al., 2008). The vegetation is characterized by shrubland of *Artemisia halondendron* and *Caragana microphylla*, with scattered trees and windbreak tree belts of *Populus* spp.

### 2.2 Experimental design, treatments, and sampling

**Figure 1:**
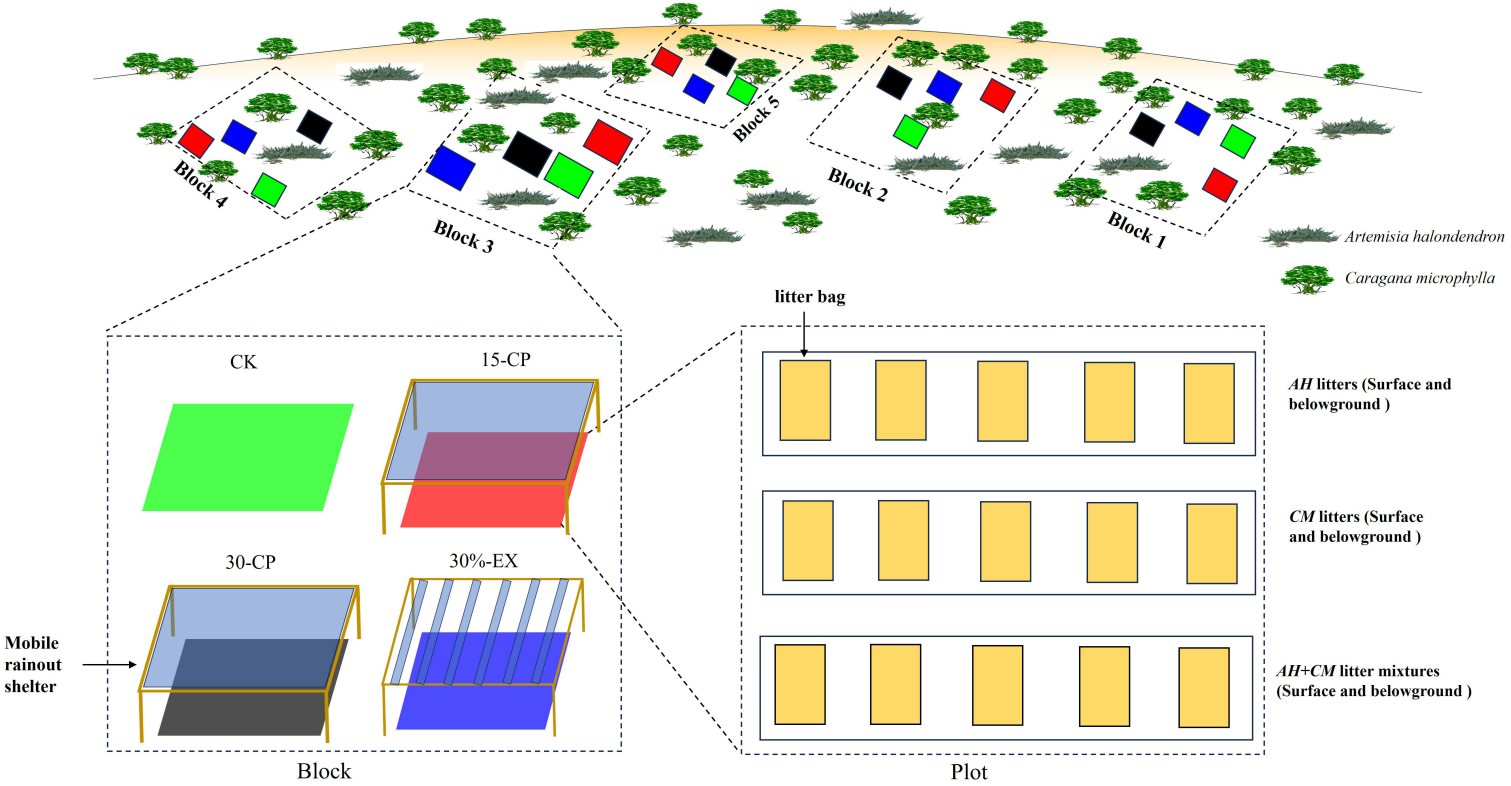
Schematic diagram of the experimental design including the block design, the distribution of litter, and the rain manipulation.

The incubation experiment was performed in a relatively flat shrubland of NDRS, using the litterbag method from May to October 2016. Twenty plots of 1.5×1.5 m^2^ were scatteredly set up in bare soil areas between shrubs with a 1-m wide buffer zone at least around each plot to avoid the shade by shrubs. The herbaceous plants in the plots were artificially pulled up to minimize soil disturbance prior to the beginning of the experiment. A random block design was used in which there were five blocks subjected to each of four rainfall repackaging treatments, including CK, 30%-EX, 15-CP, and 30-CP (**Figure 1**). CK treatment was remained naturally with ambient precipitation. 30%-EX treatment means 30% of the ambient precipitation was intercepted by mobile rainout shelters. In treatment 15-CP, the ambient precipitation was totally intercepted by mobile rainout shelters but collected in a container. When the amount of collected precipitation equal to or exceed 15 mm, it was artificially sprinkled into the soils under the corresponding shelter of 15-CP treatment. Similar method was carried out in 30-CP treatment where the amount of collected precipitation was managed to equal to or exceed 30 mm. Prior to the experiment, soils in all the blocks were wetted to 75% of field capacity to avoid any initial differences in soil moisture. Soil moisture (0 - 15 cm) was monitored about every 15 days using a portable Time-Domain Reflectometry (TDR) during the experimental period.

We performed precipitation repackaging treatments with two types of mobile rainout shelters (1.8 m × 1.8 m, 0.60 m mean height, 20° inclination), which had roofs made of transparent polycarbonate sheets for 15-CP and 30-CP treatments but V-folded transparent polycarbonate bands (with 1.8 m length and 0.09m width) for 30%-EX treatment, respectively (**Figure 1**). For the rainout shelters above the plots of 30%-EX treatment, we used six bands of V-folded transparent polycarbonate as the roof at a distance from each other of 25 cm to intercept 30% of natural rainfall. For the rainout shelters above the plots of 15-CP and 30-CP treatments, we used a 1.8 m × 1.8 m transparent polycarbonate sheet as the roof to totally intercept the natural rainfall (Yahdjian and Sala, 2002). Shelter sides remained open always to maximize air movement and minimize temperature and relative humidity artefacts. To minimize the solar radiation interception above the plots, rainout shelters were set up only before rainfall events and removed instantly after rain. Meanwhile, shelters were put on the target plots at night or on overcast days to avoid the adverse impacts of off-guard rainfall.

**Figure 2:**
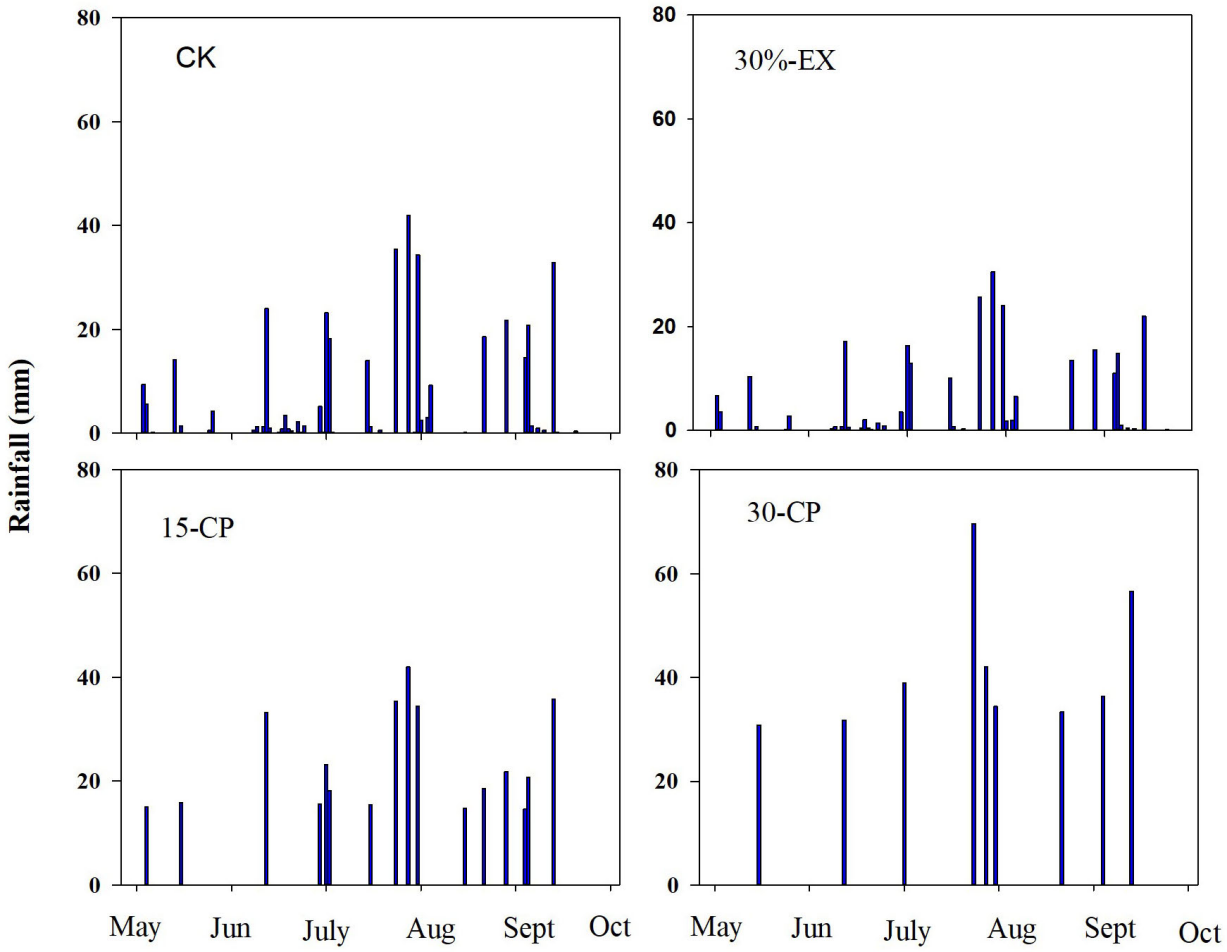
Rainfall distribution and amount in repackaging precipitation treatments during the experimental period. 15-CP, pulse size of repackaged ambient precipitation ≥ 15 mm; 30-CP, pulse size of repackaged ambient precipitation ≥ 30 mm; 30%-EX, 30% of the ambient precipitation was intercepted by rain-exclusion shelters; CK, untreated ambient precipitation.

Throughout the experimental period, ambient accumulation precipitation in the study area was 375 mm. Under repackaging precipitation treatments, incoming rainfall varied from 0.2 mm to 42 mm (46 events and 375 mm of total precipitation) in the CK treatment, from 0.1 mm to 31 mm (46 events and 262.5 mm of total precipitation) in the 30%-EX treatment, from 15 mm to 42 mm (16 events and 375 mm of total precipitation) in the 15-CP treatment, and from 31 mm to 70 mm (9 events and 375 mm of total precipitation) in the 30-CP treatment (**Figure 2**).

In October 2015, freshly senesced leaf litters *of A. halondendron* (*AH*) and *C. microphylla* (*CM*) were collected directly from the plants instead of on the soil surface. To minimize heterogeneity in litter chemistry, all plants of the two species were sampled in a small area of the shrubland. The litters were cleaned and dried at 60°C to a constant weight. Then, 15.0 g of each leaf litter of the two individual species and a litter mixture with equal proportions of the two species (*AH*-*CM* mixture, 7.5 g *AH* and 7.5 g *CM*) were enclosed in 10 × 15 cm nylon net bags. Litterbags on the soil surface were sewed with a 5-mm mesh size on the upper layer and a 1-mm mesh size on the lower layer, while belowground litterbags were sewed with a 1-mm mesh size on both layers. This combination maximized solar radiation interception aboveground and minimized soil contamination and loss of litter material (Austin et al., 2009). In May 2016, leaf litterbags of each type were buried belowground at an 8-cm depth (simulating the cover of detached litter by soil deposition in the Horqin Sand Land) and placed on the soil surfaces of the 20 plots. A total of 600 litterbags (five replicates × four treatments × three litter types × five collections × two positions) were prepared for this experiment (**Figure 1**). Litterbags were collected regularly every 30 days from May to October 2016. At each collection date, 120 bags were collected from the 20 plots for three litter types and transported to the laboratory where litters were separated from the bags, cleaned to remove any extraneous organic material, and weighed after drying at 60°C for 48 h. After the dry weight was measured, the samples were finely ground in a laboratory mill, and a portion of the litter was converted to ash to determine the ash-free dry weight of each sample. The ash-free dry mass was used for statistical analyses.

### 2.3 Chemical analysis of initial litter

Leaf litters were dried at 70°C and finely ground in a laboratory mill prior to chemical analysis. Carbon was determined by oxidation with a mixture of potassium dichromate and sulphuric acid, while nitrogen was measured by the semi-micro Kjeldahl method. P concentrations were determined colorimetrically using the ammonium molybdate method, after acid digestion (Sparks, 1996) and lignin was measured gravimetrically following extraction with 72% H_2_SO_4_ and ashing of acid detergent fiber residue (Dence, 1992). Condensed tannin concentration (% in dry matter) was extracted by adding butanol–HCl and ferric reagents to yield red anthocyanidins, and measuring absorbance at 550 nm (Porter et al., 1986). Ash was determined by a muffle furnace with litter samples burning at 550°C to a constant weight (Wang et al., 2019).

### 2.4 Data calculation and analysis

The remaining dry mass was calculated as the percentage of initial litter mass. The decomposition constant, k, was determined for all the treatments using the following equation:


$$ln\left(M_{t}/M_{0}\right)\;=\;-kt$$

where *M_0_* is the initial mass, *M_t_* is the mass remaining at given collection date *t*, and *k* represents an integrated measure of decomposition over a certain period time. We examined the non-additive effects of the *AH-CM* litter mixture by comparing the measured (MMR) and expected (EMR) litter mass remaining. EMR was calculated as the means of weighed mass remaining of individual species by their mass proportion in litter mixture for corresponding treatment. The relationship between MMR and EMR was compared with 1:1 line, with deviations from the 1:1 line indicating non-additive effects. The litter mixing effect (LME) was quantified as (*MMR*-*EMR*)/*EMR*×100%. Positive or negative LME values indicate antagonistic or synergistic effects, respectively.

One-way ANOVA was performed for multi-comparisons of the significant differences in initial litter chemistry among different litters. We used four-way ANCOVA to detect differences between precipitation repackaging, positions, litter types, collection dates, and their interactions in the remaining mass, with the block number as a covariate. For all ANOVAs, *R* ^2^ values were computed for each term by dividing the sum of squares by the total sum of squares. One-way ANOVAs were performed to detect the decomposition constant (*k*) variations for precipitation repackaging, positions, and litter types, followed by Duncan’s *post hoc* tests. Differences between MMR and EMR for the *AH-CM* litter mixture were detected by paired sample T-tests. We used three-way ANCOVA to detect the effects of precipitation repackaging, positions, collection dates, and their interactions on EMR of the *AH-CM* mixture, with the block number as a covariate. Prior to analysis, data were tested for normality using Shapiro-Wilk’s test. Response variables that did not show a normal distribution were log transformed. A level of *P* < 0.05 was accepted as significant. Data are presented as means ± standard errors. All statistical analyses were conducted using SPSS 17.0 for Windows (SPSS Inc., Chicago, Illinois, USA).

## 3 Results

**Figure 3:**
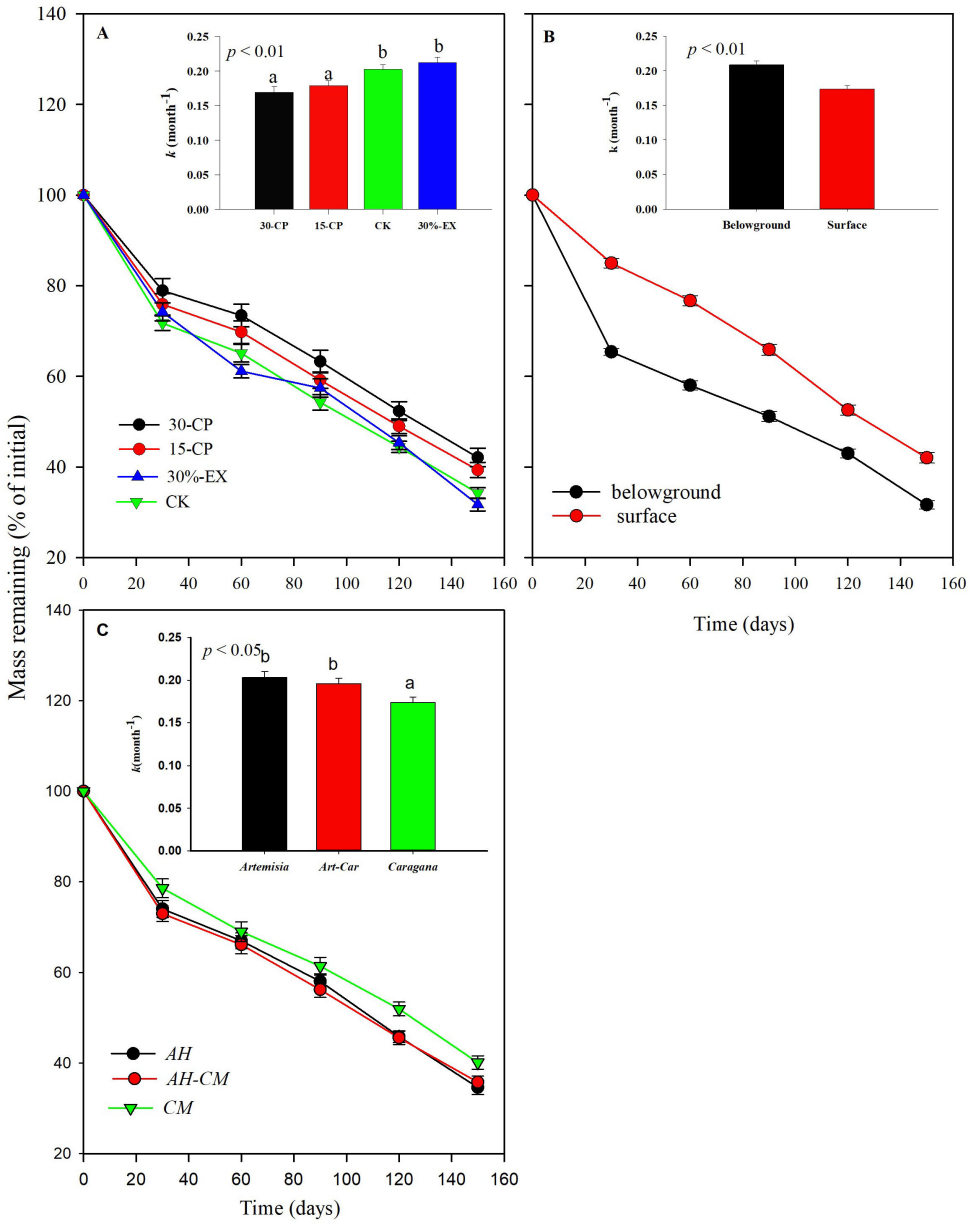
Mass of leaf litters from dominant shrubs in Horqin Sand Land remaining (mean ± SE) over time, for precipitation repackaging (**A**, *n* = 30), litter position (**B**, *n* = 60), and litter type (**C**, *n* = 40) treatments. Bars represent the decomposition constant, *k* (mean ± SE), after 5-month of incubation for a given treatment. Different lowercase letters over the bars indicate significant differences determined by least significant difference (LSD) *post hoc* comparisons at *p* < 0.05. 15-CP, pulse size of repackaged ambient precipitation ≥ 15 mm; 30-CP, pulse size of repackaged ambient precipitation ≥ 30 mm; 30%-EX, 30% of the ambient precipitation was intercepted by rain-exclusion shelters; CK, untreated ambient precipitation.

Generally, dry mass loss of leaf litter was significantly affected by rainfall repackaging, collection dates, positions, and litter types, which explained a total of 82.8% of the variance (*p* < 0.001, **Supplementary Table S1**). Over the 5-month incubation period, the mean mass loss across litter types and positions for the 15-CP and 30-CP treatments did not differ from each other but both were significantly lower than that in control treatment (**Figure 3A**). In contrast, we observed minimal effects of precipitation exclusion on mass loss. The mean mass remaining across litter types and positions was 34.2% in control plots and 31.7% in 30%-EX plots at the 5-month collection. Accordingly, no significant differences were detected in decomposition constant, *k*, between the control and 30%-EX treatments. The interaction of rainfall repackaging and litter position was also significant, indicating that the effects of rainfall repackaging on decomposition were position independent (*p* < 0.01, **Supplementary Tables S1**, **S2**). Belowground decomposition was unaffected by rainfall repackaging, while surface litter decomposition was significantly inhibited, by 4.68% and 8.07% in the 15-CP and 30-CP treatments, respectively, relative to the control treatment.

**Figure 4:**
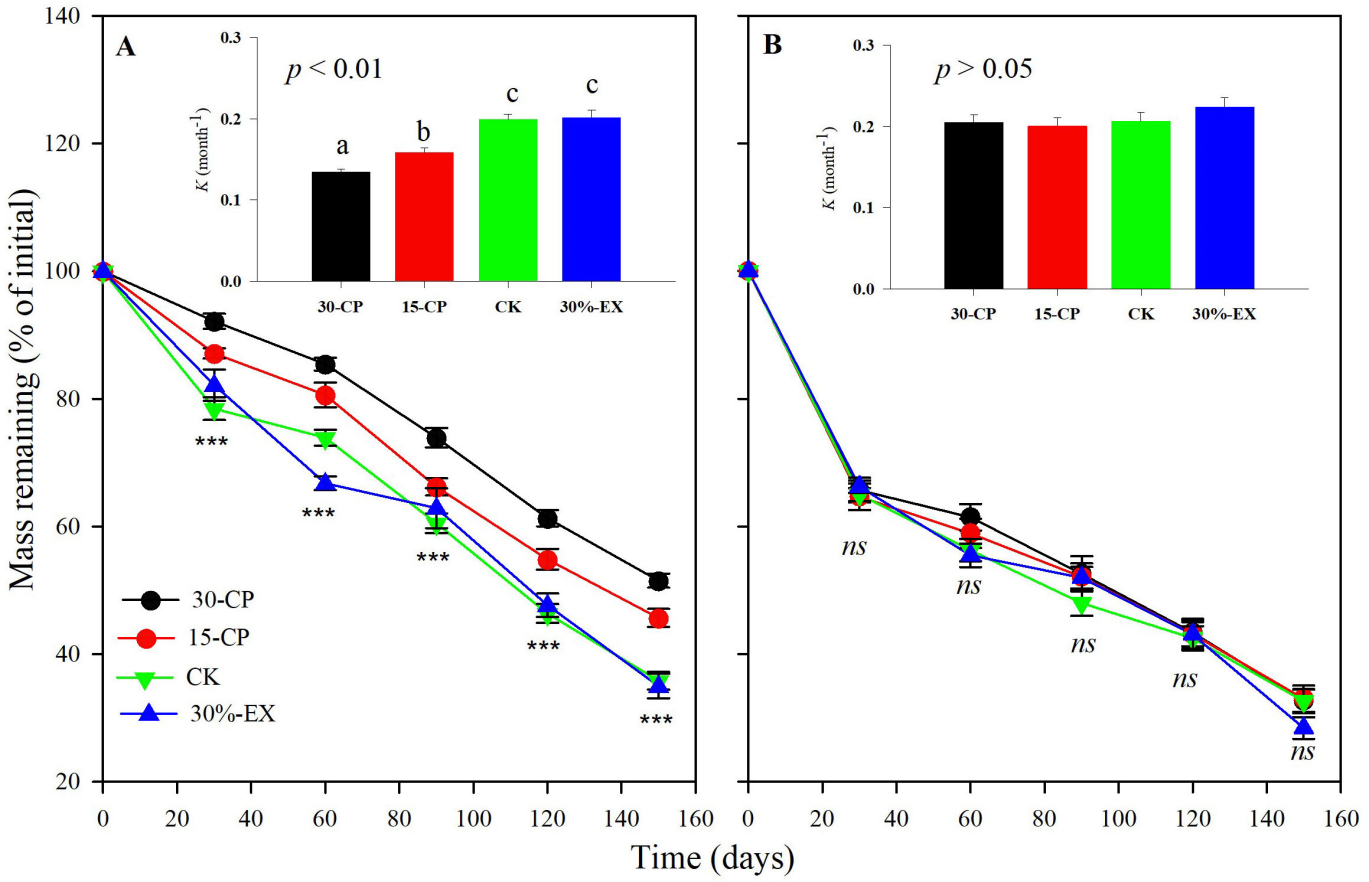
Effect of precipitation repackaging on dry mass loss of surface litters and belowground litters from dominant shrubs of the Horqin Sand Land. Mean values of mass remaining through time are *n* = 15 from **(A)** surface, and **(B)** belowground litters. Significance of main effects of precipitation repackaging treatment at a given date for surface or belowground litters is denoted as ***p < 0.001, *ns* is not significant. Bars represent decomposition constant, *k* (mean ± SE), after 5-month of incubation in precipitation repackaging treatment at different positions. Different lowercase letters over the bars indicate significant differences for least significant difference (LSD) *post hoc* comparisons at *p* < 0.05. 15-CP, pulse size of repackaged ambient precipitation ≥ 15 mm; 30-CP, pulse size of repackaged ambient precipitation ≥ 30 mm; 30%-EX, 30% of the ambient precipitation was intercepted by rain-exclusion shelters; CK, untreated ambient precipitation.

We observed that belowground leaf litters decomposed more quickly than surface litters (**Figure 3B**). The averaged mass of belowground remaining litters across litter type and rainfall repackaging treatments was 14.6% lower than that of surface litters over the 5-month incubation period. As a result, the mean decomposition constant, *k*, of belowground litters was significantly higher than that for surface litters (**Figures 4A, B**, **Supplementary Table S2**). Furthermore, interactions between litter position and collection dates were significant (*p* < 0.001, **Supplementary Table S1**), suggesting that litter decomposition rates between surface and belowground varied asynchronously with time. The mass loss of belowground litter collected at 1 month was a mean of 19.6% higher, but was 10.6% higher than those on the surface after 5-month incubation.

**Figure 5:**
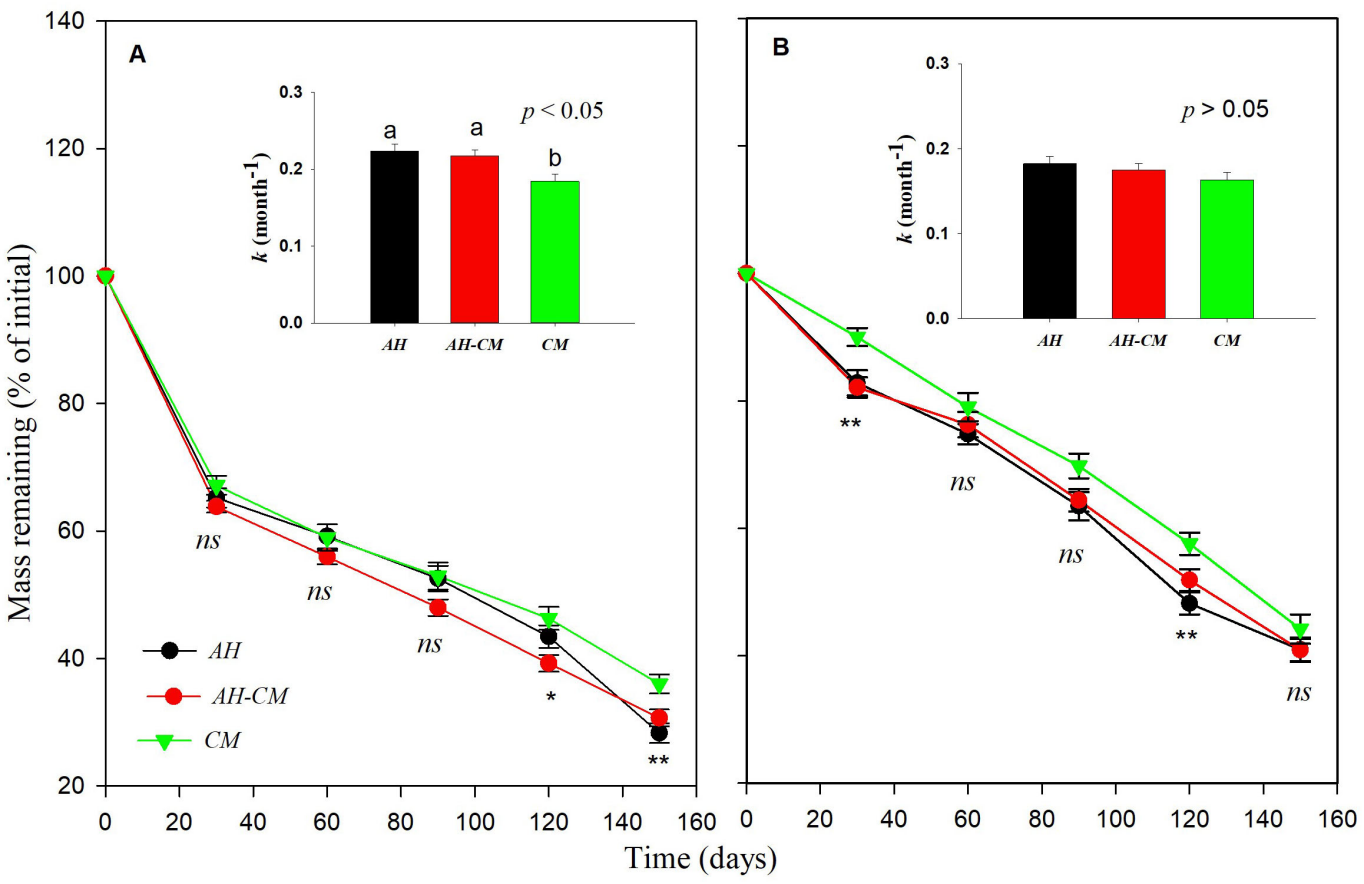
Effect of litter types on dry mass loss of surface litters and belowground litters from dominant shrubs of the Horqin Sand Land. Mean values of mass remaining through time are *n* = 20 from **(A)** surface and **(B)** belowground litters, respectively. *AH* represent leaf litter of shrub *Artemisia halondendron*, *CM* represents leaf litter of shrub *Caragana microphylla*, and *AH-CM* represents litter mixture of *A*. *halondendron* and *C*. *microphylla*. Significance of main effects of litter type treatment at a given date between surface or belowground litters is denoted as *p < 0.05, **p < 0.01, *ns* is not significant. Bars represent surface and belowground litters decomposition constant, *k* (mean ± SE), after 5-month of incubation in litter type treatments. Different lowercase letters over the bars indicate significant differences for least significant difference (LSD) *post hoc* comparisons at *p* < 0.05.

Decomposition rates were significantly different among litter types (**Figure 3C**). *CM* litter decomposed more slowly than *AH* litter. *CM* litter has higher nitrogen contents, lignin and condensed tannin content than AH litter, and lower C:N ratios than AH litter (**Table 1**). Additionally, the litter type significantly interacted with the position (*p* < 0.05, **Supplementary Table S1**). We observed that *k* values of *AH* and *AH-CM* mixture for belowground litter decomposition were significantly higher than that of *CM* (**Figure 5**), whereas no significant difference were detected in the decomposition constant, *k*, for surface litter decomposition among litter types.

**Table 1:** Initial chemical composition of the three litter types used in decomposition experiments Data are means ± SE, *n* = 5.

	*A.halodendron*	*C. microphylla*	*AH*-*CM*
C%	45.6 ± 0.13c	47.0 ± 0.04a	46.4 ± 0.1b
N%	1.9 ± 0.04c	2.5 ± 0.3a	2.2 ± 0.03b
P%	0.22 ± 0.04a	0.14 ± 0.00c	0.17 ± 0.05b
Lignin%	9.9 ± 0.3c	16.0 ± 0.21a	13.1 ± 0.13b
condensed tannin%	0.89 ± 0.02c	1.89 ± 0.01a	1.38 ± 0.03b
N:P	9.0 ± 0.2c	17.8 ± 0.2a	12.9 ± 0.3b
C:N	23.6 ± 0.5a	18.8 ± 0.2c	20.9 ± 0.3b
Lignin:N	5.1 ± 0.1c	6.4 ± 0.1a	5.9 ± 0.1b

*AH*-*CM* denote mixed leaf litter in a 1:1 ratio of *A. halodendron* and *C. microphylla*. Different lowercase letters within rows indicate significant differences (*p <*0.05) among different litter sources.

**Figure 6:**
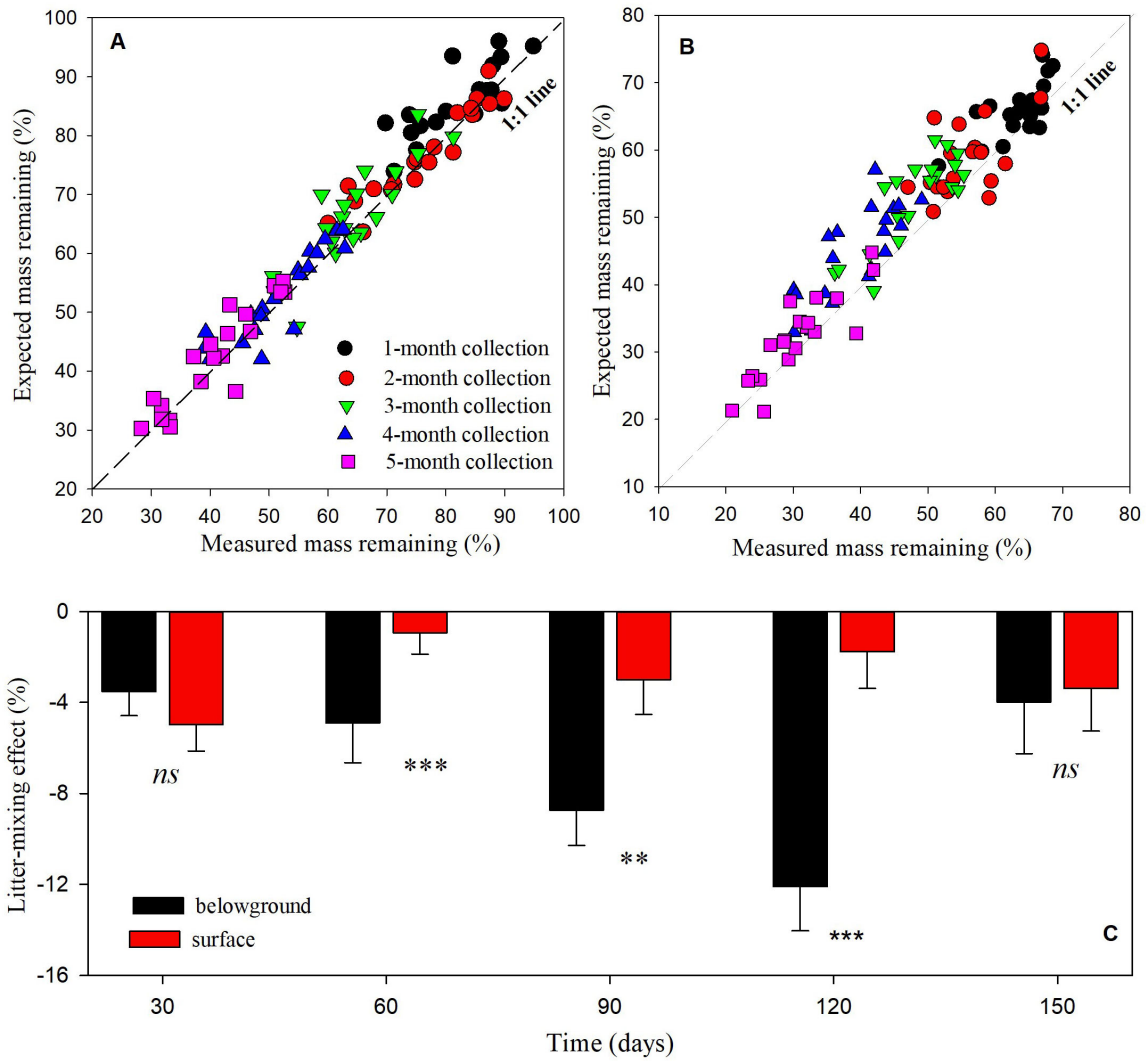
Expected vs. measured mass remaining in **(A)** surface and **(B)** belowground litters, and **(C)** mixing effects over time in mixture of shrub *AH* and *CM*. The relationship between expected vs. measured mass remaining are compared with the 1:1 line, with deviations from the 1:1 line indicating antagonistic or synergistic effects and an overlap of the 1:1 line indicating additive effects. Means of litter-mixing effects through time are *n* = 20 from surface and belowground litter mixtures. Significance of soil-covering on litter-mixing effects at a given date are denoted as **p < 0.01, ***p < 0.001, *ns* is not significant.

The measured mass remaining was significantly lower than the expected mass remaining of *AH-CM* litter mixtures as paired sample T-test (*t* = -9.46, *n* = 199, *p* < 0.001), suggesting a non-additively synergistic effect of litter mixing on decomposition. Although only 29.4% of variance was totally explained by the main effects and their interactions, ANCOVA analysis with the block number as a covariate revealed that litter-mixing effects were significantly affected by litter position, and the interaction between litter position and collection date (**Supplementary Table S3**). More synergistic effects were frequently found for the belowground litter mixture, with 84% of *AH-CM* mixtures decomposing faster than expected over the 5-month incubation. In contrast, approximately 73% of *AH-CM* mixtures on the soil surface decomposed faster than expected over the 5-month incubation. Additionally, significant differences were detected in litter-mixing effect between surface and belowground litters at 2-, 3-, and 4-month collection times (**Figure 6**).

## 4 Discussion

Litter decomposition is a fundamental process of biogeochemical cycles (Cusack et al., 2018; Vitousek, 1982), which are influenced by both extrinsic factors (temperature, precipitation and disintegrators) and intrinsic factors (litter quality) (Robinson, 2002; Luo et al., 2010). As expected, we observed significant differences in dry mass loss in leaf litter among the three litter types in the present study. With higher lignin and condensed tannin contents, *CM* litter had the lowest decomposition rate among the three litter types over the entire incubation period, suggesting that decomposition was primarily inhibited by litter recalcitrant compounds. However, litter type explained a limited amount of variance (1.53%). Regardless of collection dates which accounted for 60.33% of the variance, rainfall variability and litter position explained 3.74% and 17.22% of the variance, respectively. Although several researches suggested that litter quality plays a more important role in regulating litter decomposition than environmental factors (Aerts et al., 2003; Cornwell et al., 2008), our results indicated that extrinsic factors (e.g., precipitation and soil-covering) probably exerted a great influence on litter decomposition in the semi-arid shrubland.

### 4.1 Variation between surface and belowground decomposition

Consistent with previous studies (Austin et al., 2009; Liu et al., 2015), belowground decomposition was more rapid than surface decomposition for the three litter types studied here, indicating that soil-covering enhanced litter decomposition in the semi-arid shrublands to some certain degree. However, the result contrasts with the reports that litter decomposition on the soil surface is equal to or faster than that of soil-covered litter in drylands (McBride et al., 2023). Photodegradation was considered as the main drive to facilitate litter decomposition on the soil surface in arid regions. Regardless, soil-covering may physically abrade litter and buffers litters from temperature and moisture oscillations following rain pulses. Several studies have demonstrated that plant litters covered by mineral soil may be subject to milder temperature and moisture compared to the surface litters (Lee et al., 2014; Zhou et al., 2016). This process may facilitate the growth of bacterial decomposers by altering the microclimate around litter material, as suggested by Hewins et al. (2013), who found increases in bacterial lipid biomarkers and decrease in the relative importance of the fungal biomarker around buried litters. Consequently, a lower frequency of moisture oscillations may sustain relatively higher microbial activity, resulting in rapid decomposition rates of litters covered by soil.

Our results suggested that the differences in the magnitude of dry mass loss between surface and belowground litters varied greatly with time, with larger differences at 1-month collection (19.6% difference) and smaller differences at 6-month collection (10.3% difference). This may be attributed to poor leaching of water-soluble compounds in surface litter owing to minimal precipitation prior to the 1-month collecting date (**Figure 2**). In contrast, dried litters in mineral soil ostensibly obtained water from soil solution, which likely facilitated leaching of water-soluble compounds and, thus, may promote microbial activity. The decrease in differences between surface and belowground mass loss at the 6-month collection likely reflect compensatory leaching of water-soluble compounds and breakdown by UV radiation in surface litters relative to belowground litters (Gaxiola and Armesto, 2015).

### 4.2 Rainfall variability controls

**Figure 7:**
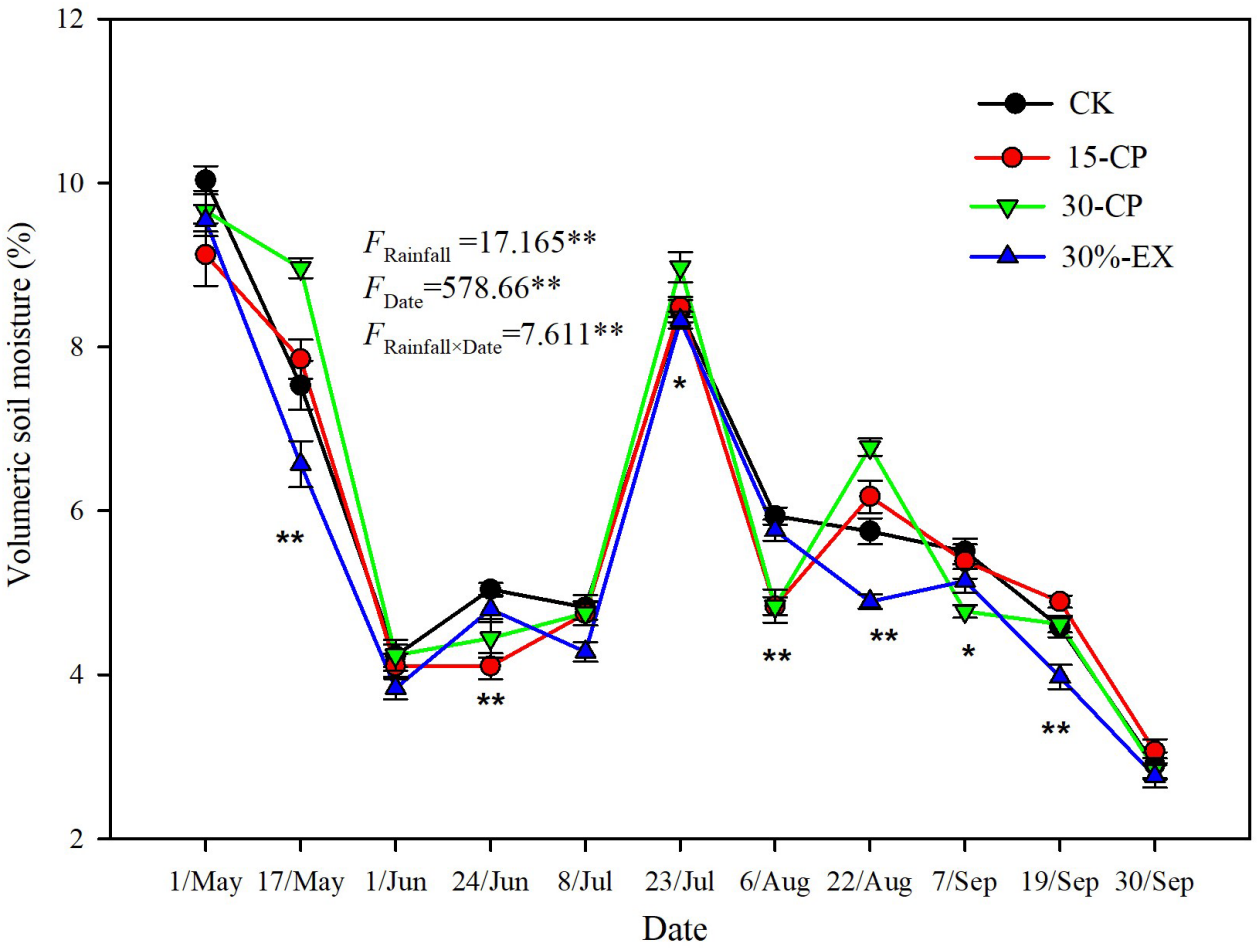
Volumetric soil moisture (0-15 cm) in repackaging precipitation treatments during the experimental period. 15-CP, pulse size of repackaged ambient precipitation ≥ 15 mm; 30-CP, pulse size of repackaged ambient precipitation ≥ 30 mm; 30%-EX, 30% of the ambient precipitation was intercepted by rain-exclusion shelters; CK, untreated ambient precipitation. Significant differences in soil moisture among the four treatments on each sampling date are indicated by *(p<0.05) and **(p<0.01).

Several previous researches have showed that the size and temporal distribution of rainfall pulses likely induce and sustain litter decomposition in drylands (Anaya et al., 2012; Li et al., 2016; Schuster, 2016). Our results confirmed that mean decomposition constant, *k*, in 15-CP and 30-CP treatments were significantly lower than that in control (**Figure 3A**), suggesting that the increase in rainfall variability strongly inhibited litter decomposition in the shrubland. Specifically, we observed that large and infrequent rainfall events inhibited surface litter decomposition relative to smaller but more frequent events for a given cumulative rainfall quantity. This result contrasts with previous findings which show that surface litter decomposition is irrespective to pulse size and frequency (Joly et al., 2017a), however, it supports the report in which small weekly pulses accelerated litter decomposition relative to larger monthly pulses for a given 25 mm cumulative precipitation (Whitford et al., 1986). Regardless, we observed insignificant differences between rainfall repackaging treatments on decomposition of soil-covered litter, which is in contrast to previous reports that showed small and large rainfall event treatments demonstrated large differences in their effects on decomposition (Austin et al., 2009; Anaya et al., 2012). Under field conditions of arid ecosystems, strong evapotranspiration usually accelerates losses of moisture from the soil surface. Topsoil is consequently subjected to frequent dry–wet cycles, whereas the underlying soil is less often influenced by rapid losses of the topsoil moisture (Austin et al., 2004). This may explain the different responses between surface and belowground decomposition to increases in rainfall variability observed in our study. In the present study, similar trends in soil moisture (0–15 cm) were detected across four rainfall treatments over the entire experiment although significant differences in soil moisture among rainfall repackaging treatments were observed on 7 of the 11 sampling dates (**Figure 7**). This results directly indicates that rainfall distribution variability alters dry–wet cycles of soil moisture, and, thus, influence microbial activity in dryland systems. However, moisture of topsoil and underlying soil are greatly modified by soil properties that influence runoff, infiltration, and water holding capacity (Lee et al., 2014). To validate this conjecture, further studies are needed to specifically clarify the response of hydrological process in the soil, and consequently microbial activity, to rainfall variability.

We observed insignificant effects of exclusion of 30% precipitation on mass loss in both surface and belowground litters, suggesting that variations in rainfall amount scarcely affected litter decomposition in the study site. The observation presented here coincided with the result that litter mass loss was not decreased by a 35% decrease in precipitation amount in a peatland (Bell et al., 2018). However, some opposing evidence suggests that litter decomposition rates are positively correlated with incoming annual precipitation in semi-arid ecosystems. Yahdjian et al. (2006) observed that the litter decomposition rates of *Stipa* sp*eciosa* were negatively correlated with the amount of intercepted precipitation in a semi-arid steppe. In the present rainfall exclusion experiments, temporal rainfall amount, but not frequency, was purposefully altered by rainout shelters. According to Borken and Matzner (2009), rainfall intensity has only a limited effect on C turnover while the duration of the dry period will influence cumulative C mineralization. Additionally, soil microorganisms have different thresholds of soil water availability, and some resistant groups may maintain vigorous activities even under conditions of low water availability (Treonis et al., 2000; Schwinning and Sala, 2004). This may explain the insignificant effects of rainfall exclusion on litter decomposition in the present study.

### 4.3 Litter-mixing effects

Some researchers have concluded that when different litters are mixed, litter that is more easily decomposed can stimulate the decomposition of litter that is less easily decomposed, and a synergistic effect is exhibited (Wardle et al., 2003; Liu et al., 2007). Consistent with theses previous results, we observed 80.5% of *AH-CM* litter mixtures had synergistic effect on litter decomposition in the present study, indicating that decomposition was often stimulated by mixing leaf litters of two dominant shrubs. Moreover, our results showed synergistic effect in belowground *AH-CM* mixture was relatively stronger than that in surface mixture, suggesting that soil-covering stimulate nonadditive effects of litter mixtures on decomposition of leaf litters. Probable differences in moisture oscillations between topsoil and the underlying soil may attribute to the stimulation of synergistic effect in belowground AH-CM mixture. Despite the difficulties in prediction, water availability has been considered as one of the predominant drivers to regulate non-additive effects of litter mixtures (Makkonen et al., 2013). Some robust evidence has demonstrated that drought reduces synergistic effects on litter decomposition (Santonja et al., 2015; Schuster et al., 2017).

In addition, our results showed that magnitudes of the synergistic effect between surface and belowground differed temporally, especially in the hotter months. Relative to the 1- and 5-month collections, LMEs were much stronger in the belowground *AH-CM* mixture than in the surface *AH-CM* mixture at 2-, 3-, and 4-month collections (**Figure 6**). Rainfall in the study site is unimodal, with a considerable amount occurring in the hotter summer months (June-August). This suggests that seasonal changes in precipitation and temperature may regulate the synergistic effect of belowground mixture litters. Large precipitation amounts along with high temperatures were considered to improve the microenvironmental conditions for litter consumers (e.g., fungi and detritivores) in soils, which in turn stimulates the litter fragments (Makkonen et al., 2013; Wu et al., 2013). This highlights the need to consider environmental conditions such as precipitation, temperature, and soil-covering when predicting the direction and magnitude of LMEs in dryland systems.

## 5 Conclusions

Our results showed that variations in rainfall amount have no effect on litter decomposition. By contrast, variations in rainfall distribution exert a great influence on litter decay process. Specifically, repackaging natural precipitation into large and infrequent rainfall events inhibited surface litter decomposition in the study. It implies that increasing rainfall variability in the current context inevitably affects organic matter turnover in dryland system. Additionally, we found that belowground litters decomposed more quickly than surface litters in the semi-arid shrubland, indicating that soil covering may buffer litter from rainfall variability by maintaining milder soil moisture and, thus, sustain microbial activity in dryland systems. There also exhibited significant litter-mixing effects in litter mixtures of two dominant shrubs, especially in the soils in the hotter months. Such results suggest that litter mixing and soil covering jointly drive litter decomposition in the context of increasing rainfall variability in distribution. In summary, these findings highlight the role of increasing rainfall variability and subsequent soil-covering or litter-mixing in driving organic matter turnover in drylands.

## Data Availability

The raw data supporting the conclusions of this article will be made available by the authors, without undue reservation.
