# Supplementary material for: Rainfall distribution variability controls surface but not belowground litter decomposition in a semi-arid shrubland
Source: Front Plant Sci. 2025 Feb 5;16:1455170. doi: 10.3389/fpls.2025.1455170 (PMC11839210; doi:10.3389/fpls.2025.1455170)
Supplement: Supplementary file 1 [file Table1.docx]

**Supplementary information**

Table S1 ANCOVA results for the effects of rainfall repackaging, little type, position, and collection date on remaining dry mass (% of initial) with the block number as a covariate

| Factor | df | %*SS* | *F* value | *P* value |
| --- | --- | --- | --- | --- |
| Block | 1 | 0.09 | 3.58 | 0.059 |
| Rainfall repackaging | 3 | 3.74 | 49.9 | **<0.001** |
| Position | 1 | 17.22 | 689.49 | **<0.001** |
| Litter type | 2 | 1.53 | 30.71 | **<0.001** |
| Collection date | 4 | 60.33 | 603.75 | **<0.001** |
| Rainfall × Position | 3 | 1.95 | 26.08 | **<0.001** |
| Rainfall × Litter | 6 | 0.02 | 0.15 | 0.989 |
| Rainfall × Collection | 12 | 0.51 | 1.71 | 0.062 |
| Position × Litter | 2 | 0.21 | 4.17 | **0.016** |
| Position × Collection | 4 | 1.37 | 13.72 | **<0.001** |
| Litter × Collection | 8 | 0.16 | 0.82 | 0.582 |
| Rainfall × Position × Litter | 6 | 0.03 | 0.17 | 0.984 |
| Rainfall × Position × Collection | 12 | 0.22 | 0.73 | 0.723 |
| Rainfall × Litter × Collection | 24 | 0.13 | 0.21 | 1.000 |
| Position × Litter × Collection | 8 | 0.32 | 1.58 | 0.127 |
| Rainfall × Position × Litter × Collection | 24 | 0.20 | 0.33 | 0.999 |

R squared=0.881

Table S2 ANCOVA results for the effects of rainfall repackaging, position, and litter type on the decomposition constant, k, with the block number as a covariate

| Factor | df | %SS | *F* value | *P* value |
| --- | --- | --- | --- | --- |
| Block | 1 | 1.92 | 3.87 | 0.052 |
| Rainfall repackaging | 3 | 16.11 | 10.84 | **<0.001** |
| Position | 1 | 16.60 | 33.5 | **<0.001** |
| Litter type | 2 | 8.52 | 8.6 | **<0.001** |
| Rainfall × Position | 3 | 7.58 | 5.1 | **0.003** |
| Rainfall × Litter | 6 | 0.14 | 0.048 | 1.000 |
| Position × Litter | 2 | 1.25 | 1.26 | 0.288 |
| Rainfall × Position × Litter | 6 | 0.80 | 0.27 | 0.949 |

R squared=0.529

Table S3 ANCOVA results for the effects of rainfall repackaging, position, and collection date on litter-mixing effects with block number as a covariate

| Factor | df | %SS | *F* value | *P* value |
| --- | --- | --- | --- | --- |
| Block | 1 | 0.04 | 0.10 | 0.751 |
| Rainfall variations | 3 | 1.01 | 0.76 | 0.521 |
| Position | 1 | 6.16 | 13.88 | <0.001 |
| Collection date | 4 | 3.60 | 2.03 | 0.093 |
| Rainfall × Position | 3 | 0.26 | 0.19 | 0.901 |
| Rainfall ×Collection | 12 | 4.14 | 0.78 | 0.673 |
| Position ×Collection | 4 | 7.05 | 3.97 | 0.004 |
| Rainfall × Position × Collection | 12 | 7.17 | 1.35 | 0.198 |

R squared=0.294
